# Acute arsine poisoning after exposure on cleaning an industrial purifier at a family-run workshop

**DOI:** 10.3389/fpubh.2025.1542879

**Published:** 2025-04-09

**Authors:** Hua Zou, Meibian Zhang, Panqi Xue, Fang Wei, Fei Li, Xinglin Fang, Xiaoming Lou, Lifang Zhou

**Affiliations:** ^1^Institute of Occupational Health and Radiation Protection, Zhejiang Provincial Center for Disease Control and Prevention, Hangzhou, China; ^2^National Institute of Occupational Health and Poison Control, Chinese Center for Disease Control and Prevention, Beijing, China

**Keywords:** arsine, occupational exposure, poisoning, workplace, hemolysis

## Abstract

We report a rare incident of acute arsine poisoning when cleaning an industrial purifier to enhance awareness for the prevention of arsine poisoning incidents in the family-run workshop. In this incident, three workers of a family-run cleaning workshop developed symptoms of headache, abdominal pain, chills, fatigue, and hematuria after cleaning an industrial purifier, with a latency period 4–6 h after exposure. Field investigations and laboratory tests were used to investigate the poisoning incident. Red blood cell counts and levels of hemoglobin, alanine transaminase, and urinary arsenic (As) increased, and urinary protein and occult blood tests indicated strongly positive results. The cleaning workshop undertook no effective measures to prevent occupational poisoning. The As concentration in flame retardants, dust on the surface of the industrial purifier, and water in the soaking pool was 269 mg/kg, 1.72 × 10^5^ mg/kg, and 6.89 × 10^3^ mg/L, respectively. This incident highlighted the association of the acute arsine poisoning during the cleaning of an industrial purifier with poor occupational health management practices. Effective measures for the prevention of the acute poisoning should therefore be undertaken.

## 1 Introduction

Arsine (arsenic trihydride [AsH_3_]) is a highly poisonous, nonirritant, colorless gas with a mild garlicky odor. On inhalation, arsine enters red blood cells (RBCs) and preferentially binds to hemoglobin to form hemoglobin peroxides that cause a series of injuries, such as intravascular hemolysis and acute renal insufficiency (1–3). The main symptoms of arsine poisoning include chills, fever, headache, fatigue, lower back pain, hematuria, and jaundiced sclera and skin. AsH_3_ has widespread application in organic synthesis and production of semiconductors, and AsH_3_ leakage during the production process can induce occupational poisoning (4, 5). Moreover, occupational AsH_3_ poisoning often occurs in the metallurgical industry because AsH_3_ is readily produced when minerals containing arsenic (As) react with acids during the smelting, processing, and storage stages, or when their metal slag gets wet (6–8). However, AsH_3_ poisoning is rarely reported from the cleaning or maintenance industries. Herein, we report an acute AsH_3_ poisoning case in a family-run cleaning workshop during the cleaning of an industrial purifier.

## 2 Case report

On May 12, 2019, three workers from a family-run cleaning workshop dismantled an industrial purifier in one of the production lines of a factory that produces new materials and shipped it back to the cleaning company. At approximately 13:00 on May 13, 2019, the three workers soaked and cleaned the industrial purifier and completed their task around 15:00. By 17:00, two workers developed abdominal pain, diarrhea, chills, fatigue, vomiting, and “soy sauce-colored” urine. The third worker developed similar symptoms at 19:00. The three workers were initially treated either at the local community health service center or a county hospital. The patients’ condition deteriorated despite treatment, and they were transferred to a poisoning treatment center at the provincial hospital by 17:00 on May 14, 2019. One of the patients died, and the other two recovered.

### 2.1 Clinical manifestations

Table 1 lists the clinical manifestations of the patients. Three male patients in the age range 51–62 years developed clinical symptoms, such as headache, abdominal pain, chills, fatigue, and hematuria, following a 4–6 h latent period after poisoning. In all three patients, the RBC counts increased above the upper limit of the reference range, and the urinary occult blood and protein tests were strongly positive. In two of the patients, the hemoglobin and alanine transaminase levels increased. Following their transfer to the provincial center for poisoning treatment, their urine samples were tested using a toxicological method based on the Chinese standard WS/T 474–2015, which specifies the determination of arsenic in urine by hydride generation atomic fluorescence spectrometry. This standard outlines a reliable procedure for measuring arsenic levels in urine through digestion of the sample with acid mixtures, reduction of arsenic compounds to arsine gas, and subsequent detection via atomic fluorescence spectrometry. The result indicated significantly increased urinary As concentrations compared with the As reference value.

**Table 1:** Clinical manifestations and progression of acute poisoning in the three patients.

No	Age (y)	Latency period (h)	Symptom onset and progression			Clinical signs						
			Time	Symptoms	Medical treatment	RBC (×10^12^)	Hemoglobin (g/L)	UOB	UP	ALT (IU/L)	Urine A_S_ (μg/L)	Outcomes
1	51	4	17:00 on May 13th	Abdominal pain, diarrhea, chills, “tawny urine”	Symptomatic treatment at a local community health center							
			7:00 on May 14th	Shortness of breath	Treatment against hemolysis at a county hospital	3.25	98	+++	+++	−	295	Dead
			17:00 on May 14th	Continued to be worse	Arsine poisoning treatment at a provincial hospital							
2	62	4	17:00 on May 13th	Fatigue and chills	None							
			20:00 on May 13th	“Soy sauce”-colored urine	Medical treatment for chills							
			7:00 on May 14th	Continued to worsen	Treatment against hemolysis at a county hospital	4.16	135	+++	+++	140	157	Recovered
			17:00 on May 14th	Continued to worsen	Arsine poisoning treatment at a provincial hospital							
3	55	6	17:00 on May 13th	No symptoms	None							
			19:00 on May 13th	Headache, chest tightness, vomiting, abdominal pain, chills, high fever, “soy sauce”-colored urine	Treatment against hemolysis at a county hospital	3.36	104	++	+++	101	304	Recovered
			17:00 on May 14th	Continued to be worse	Arsine poisoning treatment at a provincial hospital							

RBC, red blood cell; UOB, urine occult blood; UP, urine protein; ALT, alanine transaminase.

### 2.2 Field investigations

**Figure 1:**
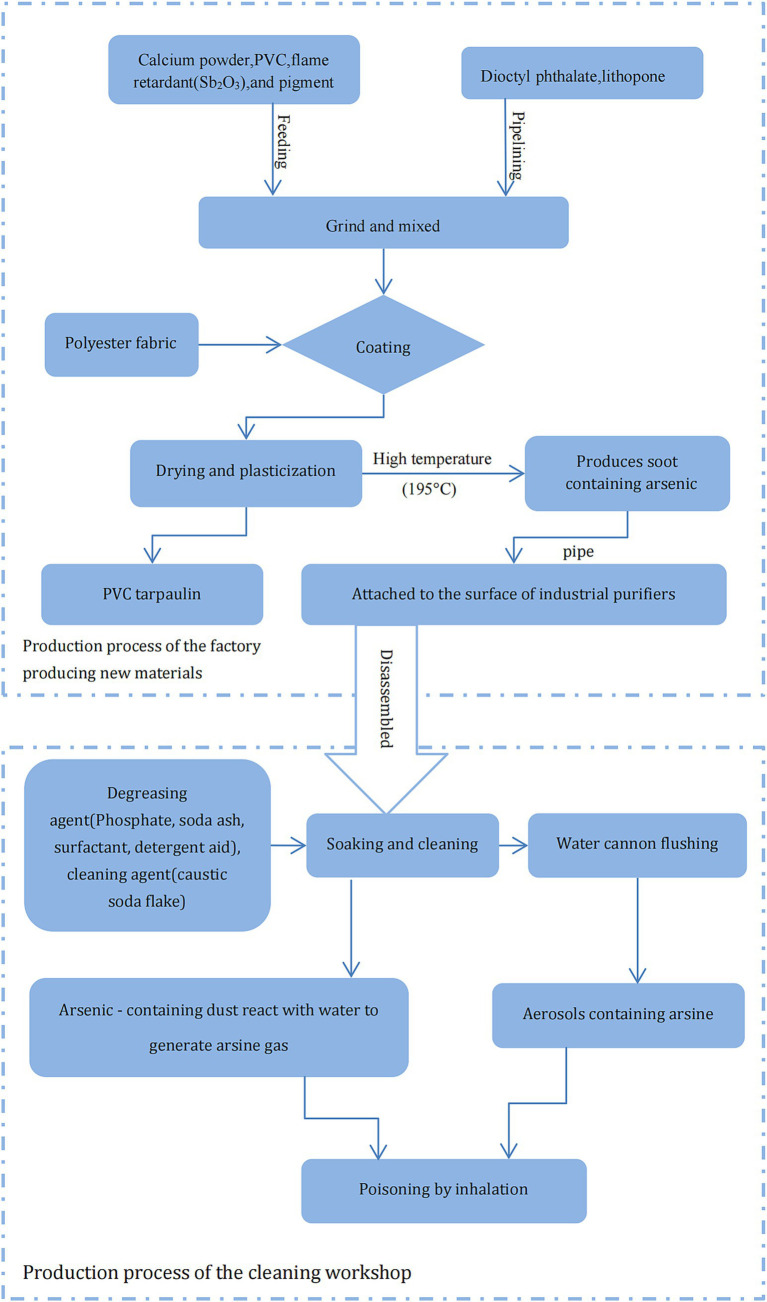
Schematic representation of the production process of relevant enterprises and potential cause of arsine poisoning.

Two factories were involved in the acute exposure: the factory producing new materials and the workshop that undertook the cleaning of the industrial purifier. The factory producing new materials is a small-size enterprise. The cleaning workshop is set on the first floor, the second floor is the living room for the employer and the employee. Details of the production processes and raw materials used by the factories are illustrated in Figure 1. Raw materials, including flame retardants (e.g., Sb_2_O_3_), were mixed and ground to produce coating materials, which were then sprayed with a coating machine to coat the polyester fabric and then transferred to an oven for drying and plasticization at 195°C; PVC tarpaulin was produced on cooling. The fumes generated during the coating and drying process adhered to the surface of the metal filter after being sucked in by the fan. At the cleaning workshop, the disassembled industrial purifier was soaked in a pool with degreasing and cleaning agents and then surface cleaned by water cannons. The local exhaust ventilation (LEV) or general ventilation system was not installed in the family-run cleaning workshop, and the three cleaning workers did not wear any effective personal protective equipment (PPE) during the cleaning operation. Furthermore, the company had not formulated any emergency plans for acute poisoning and was not equipped with any emergency rescue facilities.

### 2.3 Sampling and testing

Air samples for AsH_3_ were collected and tested 24 h after the cleaning operation; the results showed that AsH_3_ concentrations were below the detection limit. Flame retardants, dust on the surface of another industrial purifier used in the same production line, and water in the soaking pool were tested by atomic absorption spectrometry to determine As concentrations, following the standard reference GB/T 7686–2016. This standard specifies a general method for determining arsenic content of chemical products using atomic fluorescence spectrometry. The As levels of the above mentioned three materials were 269 mg/kg, 1.72 × 10^5^ mg/kg, and 6.89 × 10^3^ mg/L, respectively.

## 3 Discussion

Arsine is the most acutely toxic form of arsenic and one of the major industrial causes of sudden extensive hemolysis. It can combine with hemoglobin within the RBCs, causing destruction or severe swelling of the cell, rendering it nonfunctional. In this case, the three patients were all workers in the same workshop and at the same operating post. All the workers developed the disease within 6 h and had the same clinical symptoms such as dizziness, headache, fatigue, chills and “soy sauce color” urine. After 24 h, urinary As concentrations significantly increased, and biochemical indicators showed that the patients had hemolysis and liver and kidney damage. These typical clinical manifestations of occupational acute AsH_3_ poisoning were in agreement with relevant reports. Lee et al. (3) reported that in a metal smelter, three workers developed clinical symptoms (e.g., nausea, vomiting, gross hematuria, and decreased hemoglobin) 2 h after cleaning metal residue with sulfuric acid, which was later proved to be caused by acute arsine poisoning. Qin et al. (9) analyzed 24 cases of acute arsine poisoning and found that the latency period of poisoning was between 0.5 and 4 h, and hospitalization was between 8 and 24 h after onset; the symptoms included dizziness, fatigue, chest tightness, lumbago, nausea, vomiting, and soy sauce-colored urine.

In several industrial processes, such as electrolysis, smelting, and disposal of wet slag, AsH_3_ can be readily generated even if there are trace amounts of arsenic compounds (10, 11). Conrad et al. (12) reported that three workers were poisoned while using a jackhammer to remove slag from ladles. To reduce dust, water was sprayed on the slag, and the water reacted with the arsenide of the alkali metal, generating arsine. Figure 1 demonstrates the potential cause of the AsH_3_ poisoning in this incident. Stibnite (Sb_2_S_3_) is the primary raw material used in the production of the flame retardant Sb_2_O_3_. During the industrial processing of stibnite, arsenic is often found as a coexisting element with antimony due to their similar geochemical behaviors (13). The production process of Sb_2_O_3_ from stibnite typically can generate arsenic-containing by-products. Additionally, the refining and purification processes may not completely remove arsenic, resulting in residual As_2_O_3_ in the final product Sb_2_O_3_ (14). Therefore, Arsenic content detected from the flame retardant Sb_2_O_3_ used at the new materials factory probably presented as As_2_O_3_, which possibly decomposed into elemental As at high temperatures and accumulated on the surface of the industrial purifier on being vented through an LEV. During soaking, cleaning, and water-cannon flushing, the As on the purifier surface could have been hydrolyzed to AsH_3_ on exposure to water. The AsH_3_ thus produced could have reached very high concentrations in the poorly ventilated cleaning workshop due to the poor management of occupational health conditions. Therefore, based on the typical clinical manifestations, field investigation, and laboratory tests, the critical presentation was attributed to acute AsH_3_ poisoning, in accordance with the Chinese occupational health standard GBZ 44–2016, which specifies the diagnosis and management principles for occupational acute arsine poisoning.

The acute arsenide poisoning was mainly due to the lack of awareness of occupational disease control by the employers of the family-run workshop, and the absence of necessary facilities preventing acute poisoning in the work site, resulting in the failure of timely discharge of the arsenide generated in the operation process and thus, an increased arsenide concentration in the workplace. In addition, workers, employers, and clinicians at the grassroots level are often unaware of acute poisoning; therefore, diagnosis and treatment of acute poisoning are often ignored and delayed. Arsine is often not a raw material or product, but an intermediate product or waste gas in the production process (15, 16). It is colorless and odorless, and the poisoning caused by it is often neglected and delays the diagnosis and treatment of patients. In this case, two patients went to county hospitals for treatment 14 h after poisoning but did not receive timely, targeted treatment, and their clinical condition continued to worsen in the early stage of poisoning.

## 4 Conclusion

These emergency cases of occupational acute AsH_3_ poisoning were associated with poor occupational health management during the cleaning of an industrial purifier. Based on the lesson, the following measures should be undertaken to prevent similar poisoning incidents: strengthening the occupational health knowledge through training employers, workers and clinicians at the grassroots level, and improving their awareness of acute arsenic poisoning; strengthening the local government’s supervision of small-sized enterprises and family-run workshops; an exposure control strategy should be developed: the arsenic impurity test for flame retardants should be conducted to ensure As-free status prior to purchasing raw materials; the soaking or cleaning process should be undertaken in contained workspaces equipped with LEV; and developing an emergency response plan for acute arsenic poisoning; and wearing effective PPEs for cleaning workers.

## Data Availability

The raw data supporting the conclusions of this article will be made available by the authors, without undue reservation.
